# Conceptualization, Operationalization, and Measurement of Adherence to Patient-Facing Digital Health Applications: Protocol for a Scoping Review

**DOI:** 10.2196/95287

**Published:** 2026-07-08

**Authors:** Ludovico Cavallaro, Philipp Graf, Josephine Verona Konrad, Julia Zielke, Jan C Zoellick, Danielly de Paula, Ariel Dora Stern

**Affiliations:** 1 Hasso Plattner Institute, Digital Engineering Faculty, University of Potsdam, Prof.-Dr.-Helmert-Str.2-3, Potsdam, Brandenburg, 14482, Germany, +49 33155093950; 2 Charité — University Medical Center Berlin, corporate member of Freie Universität Berlin and Humboldt-Universität zu Berlin, Berlin, Germany; 3 Cork University Business School, University College Cork, Cork, Ireland; 4 Windreich Department of Artificial Intelligence and Human Health, Icahn School of Medicine at Mount Sinai, New York, NY, United States

**Keywords:** adherence, patient-facing, digital health applications, conceptualization, definition, operationalization, measurement, PRISMA, Preferred Reporting Items for Systematic Reviews and Meta-Analyses

## Abstract

**Background:**

Medical adherence is traditionally defined as the extent to which a person’s behavior corresponds with agreed-upon recommendations from a health care provider. The rapid diffusion of patient-facing digital health applications (DHAs) challenges the direct applicability of this framework to software-mediated interventions. Unlike pharmacological treatments, DHAs automatically generate detailed usage data but lack a broadly accepted standard for determining which specific data points and usage patterns actually constitute “adequate” use across a broad variety of medical indications and population groups. In parallel, DHAs are becoming increasingly embedded in formal regulatory and reimbursement pathways. A notable example is Germany, where recent reforms plan to link at least 20% of reimbursement for *Digitale Gesundheitsanwendungen*—formally regulated and reimbursed DHAs—to performance-related indicators that implicitly operationalize adherence as a determinant of reimbursable performance. Despite this growing policy relevance, adherence in patient-facing DHAs remains conceptually fragmented and methodologically heterogeneous.

**Objective:**

This scoping review aims to map how adherence is conceptualized, operationalized, and measured in the context of patient-facing DHAs, and to explore whether these approaches differ between applications embedded within formal regulatory and/or reimbursement frameworks and those operating outside such settings.

**Methods:**

This scoping review follows the Joanna Briggs Institute methodology and will be reported in accordance with the PRISMA-ScR (Preferred Reporting Items for Systematic Reviews and Meta-Analyses extension for Scoping Reviews) guidelines. PubMed (MEDLINE), Scopus, and Web of Science will be searched for scientific literature published between 2020 and 2025, inclusive. Machine learning–assisted title and abstract screening will be performed using ASReview, following a sampling-based stopping criterion. Three independent reviewers will conduct screening and data extraction. Relevant gray literature will be identified through targeted searches.

**Results:**

Database searches identified 15,403 records (5469 from PubMed/MEDLINE, 6198 from Scopus, and 3736 from Web of Science), which were reduced to 7052 unique records after automated deduplication using Zotero (Corporation for Digital Scholarship). The full results are intended to be submitted to a peer-reviewed journal by the end of 2026.

**Conclusions:**

Conducted as part of the DEKODE *(Digital Health Adhärenz: Konzept und datenbasierte Evidenz*) project funded by the German Federal Joint Committee’s Innovation Fund, this review contributes to the development of a conceptual and empirical adherence framework for patient-facing DHAs. Although DEKODE is primarily oriented toward the German *Digitale Gesundheitsanwendungen* context, this review applies no geographical restrictions, reflecting the value of establishing a broad evidence base before narrowing the focus to country-specific settings and rendering findings relevant for understanding DHA adherence in all geographies. By clarifying how adherence is currently conceptualized, operationalized, and measured, the review will promote more consistent and context-specific generation and interpretation of adherence-related evidence and support evidence-based policy discussions, international harmonization of evidence standards for DHAs, and health economic evaluations in digital health.

## Introduction

### Background

Medical adherence is traditionally defined by the World Health Organization (WHO) as the extent to which a person’s behavior, including taking medication, following a diet, and executing lifestyle changes, corresponds with agreed-upon recommendations from a health care provider [1]. This definition marked a deliberate shift from earlier, medication-centered views by emphasizing 2 core dimensions. First, adherence encompasses a broad range of therapeutic behaviors, including self-management activities, follow-up attendance, and lifestyle modification. Second, it presupposes a collaborative relationship between patients and health care providers, in which treatment plans are negotiated rather than unilaterally prescribed, and adherence is actively discussed, supported, and monitored over time [1,2].

This behavioral and relational foundation has proven conceptually robust in conventional health care settings, particularly where treatment regimens are clearly defined in terms of dosage, frequency, and duration. However, the rapid diffusion of patient-facing digital health applications (DHAs) challenges the direct transferability of this framework to software-mediated interventions [3-5]. Unlike pharmacological treatments, which are typically characterized by a defined therapeutic dose and schedule, DHAs rarely have a universally accepted or empirically established notion of “optimal use” [4]. Although DHAs generate detailed usage data by design, the mere availability of digital logs (metadata) capturing frequency, duration, sequence, and intensity of use does not intrinsically define what counts as adequate adherence. In the absence of a shared reference standard for therapeutic “dose,” these usage metrics are analytically rich but conceptually underdetermined [3].

To address this gap, several authors have proposed the concept of *intended use*, defined as using the technology in the way it was designed to be used, and for a duration or intensity presumed necessary to achieve benefit [3,4,6-8]. This concept acknowledges that different DHAs may require distinct patterns of use to produce health outcomes. However, intended use thresholds are rarely standardized, consistently justified, or empirically validated, and often remain implicit, intervention-specific, or defined post hoc [4,7]. Consequently, adherence metrics across DHAs are highly inconsistent, limiting comparability between studies and complicating the generation and interpretation of adherence-related evidence in clinical, economic, and regulatory contexts.

These conceptual and methodological challenges continue to grow in relevance as DHAs increasingly transition from experimental settings into standardized health care pathways. A notable case is Germany, where the Digital Healthcare Act (*Digitale-Versorgung-Gesetz*), enacted in November 2019, established a regulatory and reimbursement framework under which patient-facing, lower-risk digital medical devices (*Digitale Gesundheitsanwendungen* [DiGA]) can be prescribed by physicians and reimbursed within the statutory health insurance system following assessment by the Federal Institute for Drugs and Medical Devices (*Bundesinstitut für Arzneimittel und Medizinprodukte*) [9,10].

Within this framework, recent legislative reforms elevate the relevance of adherence by mandating that, from 2026 onwards, at least 20% of DiGA reimbursement will be linked to performance-related indicators. Under the Digital Act (*Gesetz zur Beschleunigung der Digitalisierung des Gesundheitswesens*), performance is to be assessed using measures such as the duration and frequency of use, patient satisfaction, and patient-reported health status during DiGA use. Although adherence is not defined as a stand-alone endpoint, these criteria rely directly on adherence-related usage patterns and implicitly operationalize adherence as a determinant of reimbursable performance [11].

In this evolving landscape, adherence becomes a regulatory and economic parameter with direct implications for reimbursement and market access. Yet, the underlying construct remains conceptually unsettled and methodologically heterogeneous. Existing reviews [3,4,6,7,12,13] have examined adherence within specific clinical domains, explored its association with intervention outcomes, identified determinants of adherence, and analyzed how intended use has been operationalized in electronic health technologies. However, none has provided a comprehensive and comparative mapping of how adherence is currently conceptualized, operationalized, and measured in contemporary patient-facing DHAs, nor assessed how these approaches align with the emerging regulatory and reimbursement frameworks in which such applications are increasingly embedded.

### Objectives

Given the conceptual ambiguity, methodological heterogeneity, and increasing policy relevance of adherence in digital health interventions broadly, and in DHAs in particular, this scoping review pursues 3 interrelated objectives:

- Clarify the conceptual foundations of adherence in patient-facing DHAs by identifying how the construct is defined and distinguished from related concepts.
- Examine how these conceptualizations are translated into operational definitions and measurement strategies used to capture adherence-related behaviors.
- Explore contextual variations in these approaches, particularly by comparing patient-facing DHAs embedded within formal regulatory and reimbursement frameworks with those operating outside such settings.

## Methods

### Study Design

A scoping review was selected as the most appropriate methodological approach for addressing the objectives of this study. According to Munn et al [14], scoping reviews are particularly appropriate when the objectives are to clarify key concepts and definitions in the literature, identify key characteristics or factors related to a concept, and examine emerging evidence in fields where the conceptual and methodological landscape is still insufficiently settled to support a more focused systematic review. All three of these methodological indications apply directly to this review. In line with this methodological approach, this protocol has been developed using an adapted application of the PRISMA-P (Preferred Reporting Items for Systematic Review and Meta-Analyses—Protocols) guidelines (Checklist 1) [15]. In the absence of a dedicated, stand-alone reporting guideline for scoping review protocols, PRISMA-P items are selectively applied and tailored to reflect the objectives and methodological features of scoping reviews, in line with recommendations from the Joanna Briggs Institute [16]. This protocol was not registered in PROSPERO because scoping reviews are not currently accepted by the registry. To ensure transparency and prospective documentation, the protocol was instead registered on the Open Science Framework. Any deviations from the registered protocol will be documented through updates to the Open Science Framework registration and reported in a supplementary document accompanying the final review.

The conduct of the review will follow established scoping review methodology [17], and the reporting of the completed review will adhere to the PRISMA-ScR (Preferred Reporting Items for Systematic Reviews and Meta-Analyses Extension for Scoping Reviews) guidelines (Checklist 2) [18], ensuring transparency, reproducibility, and consistency in reporting.

### Eligibility Criteria

#### Overview

Eligibility criteria for this scoping review are defined according to the Population, Concept, and Context framework, in line with the Joanna Briggs Institute’s guidance for scoping reviews [16]. Given that this review examines adherence within a category of digital health technologies rather than within specific participant groups, the “Population” element of the Population, Concept, and Context framework is reformulated as “Technology” to reflect the primary unit of analysis.

#### Technology

The technology of interest consists of patient-facing DHAs. For the purpose of this review, these are defined as digital applications primarily intended for direct use by patients and designed to prevent, monitor, manage, or treat health conditions, or to promote health-related behavior change. Eligible applications include the following:

- Preventive or health-promotion applications with an explicit health objective (eg, behavior change apps targeting smoking cessation or physical activity improvement).
- Symptom tracking and monitoring applications used within or outside formal care pathways (eg, migraine or asthma tracking apps).
- Disease-management applications for both chronic and acute conditions supporting ongoing care (eg, diabetes apps requiring regular glucose logging and feedback).
- Digital therapeutics delivering structured therapeutic content or programs (eg, app-based cognitive behavioral therapy for insomnia or depression).
- Digital rehabilitation or hybrid care applications combining monitoring and therapeutic interaction (eg, cardiac rehabilitation apps integrating activity tracking and clinician check-ins).

Applications may be delivered via mobile devices, web-based platforms, and/or other digital interfaces (eg, virtual reality headsets), provided that active patient interaction constitutes a core component of the intervention.

Applications solely providing general health information, administrative functions, or nonhealth-related wellness content without a stated health objective associated with a specific medical condition, set of conditions, or diagnosis will be excluded. Applications designed exclusively for use by health care professionals, caregivers, or health system administrators will also be excluded unless patient use is explicitly examined. Purely technical tools, background data infrastructures, or repositories without a patient-facing interface will not be eligible. No restrictions will be applied based on disease area, clinical indication, or target patient population.

#### Concept

The core concept of interest is adherence to patient-facing DHAs. This review builds on the traditional WHO definition of medical adherence as behavior corresponding with agreed-upon recommendations from a health care provider [1], while recognizing that the transferability of this behavioral and relational framework to digital health contexts is conceptually challenging. Given this ambiguity, the review does not impose a prescriptive definition of adherence.

Studies will be eligible if they report either of the following in relation to a patient-facing DHA: (1) an explicit conceptual definition of adherence or (2) an operational or measurement strategy that quantifies usage behavior relative to an intended or recommended pattern of use.

All study designs will be considered eligible. Systematic reviews and meta-analyses identified during screening will not undergo data extraction, as the unit of analysis for this review is the primary study. However, their reference lists will be screened to identify additional eligible primary studies through backward citation tracking. Conference abstracts, editorials, commentaries, and study protocols will be excluded due to insufficient detail.

#### Context

The context of interest is the use of patient-facing DHAs within health care and health-related settings, including preventive, diagnostic, monitoring, therapeutic, or rehabilitative contexts.

The review is not restricted to any single country or health care system. Studies conducted in any geographical location and health care setting will be eligible.

No language restrictions will be applied, in line with the global scope of the review. Studies published in English, French, German, Italian, and Portuguese will be assessed directly, reflecting the language competencies of the research team. For studies published in other languages, 2 large language model–based tools (ChatGPT from OpenAI and Claude by Anthropic) will be used to support translation. The outputs generated by the 2 tools will be compared to assess interpretative consistency. Where discrepancies between translations prevent confident interpretation of the text, corresponding authors will be contacted for clarification. Details of the translation process, including the artificial intelligence models used and the generated outputs, will be reported in the supplementary material of the final review.

The review will focus on literature published from 2020 to 2025 inclusive, a period selected for 2 key reasons. First, given the rapid pace of technological innovation in digital health, earlier studies may reflect technological configurations, usage patterns, and analytical practices that differ substantially from current applications. Second, it encompasses the emergence and consolidation of national frameworks for the assessment, validation, regulation, reimbursement, and integration of such applications across diverse health care systems. Since 2020, initiatives including DiGA Fast Track (Germany), PECAN (*Prise en Charge Anticipée Numérique*, France), Early Value Assessment (United Kingdom), and the mHealth validation pyramid (Belgium) have shaped how DHAs are assessed and integrated into clinical pathways. By focusing on this contemporary period, the review ensures that captured conceptualizations, operationalizations, and measurement approaches of adherence reflect current technological, regulatory, and reimbursement frameworks.

### Information Sources

The literature search will use a combination of structured database queries and citation-based retrieval techniques. Electronic searches will be conducted in PubMed (MEDLINE), Scopus, and Web of Science. These databases were selected to provide broad and complementary coverage of biomedical, interdisciplinary, health services, and digital health–related literature relevant to patient-facing DHAs and adherence research, consistent with prior systematic reviews [3,4].

Backward citation tracking, defined as the systematic screening of reference lists, will be applied to all included studies to minimize the risk of overlooking relevant studies.

In addition to peer-reviewed literature, targeted searches of gray literature will be conducted through Google and relevant organizational websites, including those of regulatory authorities (eg, the European Commission and the US Food and Drug Administration), international organizations (eg, the WHO and the Organisation for Economic Co-operation and Development), and industry associations (eg, the National Association of Digital Health Care, Germany, and MedTech Europe, Belgium). The same conceptual keywords and overall eligibility framework used for the scientific literature searches will also guide the identification and selection of gray literature sources.

### Search Strategy

The search strategy has been developed iteratively by the research team, drawing on prior systematic reviews in this context [3,4,6,7]. The search strategy is structured around two conceptual blocks combined using Boolean operators: (1) adherence and adherence-related constructs, and (2) DHAs.

The first block includes “adherence” and related terms, which are often used interchangeably in the literature, to maximize search sensitivity.

The second block captures DHAs using terminology commonly used in the literature. The term “patient-facing” was not incorporated into the search query, as it is not consistently used in indexing or titles and could unduly restrict retrieval. Restriction to patient-facing applications will be applied during the screening process. Database-specific syntax and controlled vocabulary were used where appropriate.

The complete search strategies, as implemented in each database, are reported in Textbox 1, which presents the full queries for PubMed (MEDLINE), Scopus, and Web of Science.

Textbox 1. **Search query for PubMed, Scopus, and Web of Science.**

**Search block 1 (all databases)**

- (adheren* OR compliance OR engagement OR retention OR dropout* OR attrition)

**Search block 2 (all databases)**

- ("digital therap*" OR DTx OR "digital health application*" OR "digital health intervention*" OR "mobile health" OR m-health OR mhealth)

**Publication year (all databases)**

- 2020-2025

**PubMed (MEDLINE) [tiab]**

- Search blocks 1 and 2 applied to title and/or abstract fields
- MeSH term: Patient Compliance

**Scopus [Title-Abs-Key]**

- Search blocks 1 and 2 applied to Title, Abstract, and Keywords
- Source type: Journals
- Document type: Excluding trade publications (tb)

**Web of Science [Topic]**

- Search blocks 1 and 2 applied to Topic fields
- Database subset: Current Contents Connect

### Study Records

#### Data Management

All records retrieved from the databases will be imported into Zotero (Corporation for Digital Scholarship), which will be used as the reference management software for record storage, organization, and duplicate removal [19]. After deduplication, the resulting set of unique records will be exported to a structured Microsoft Excel spreadsheet for title and abstract screening.

#### Selection Process

**Figure 1.**
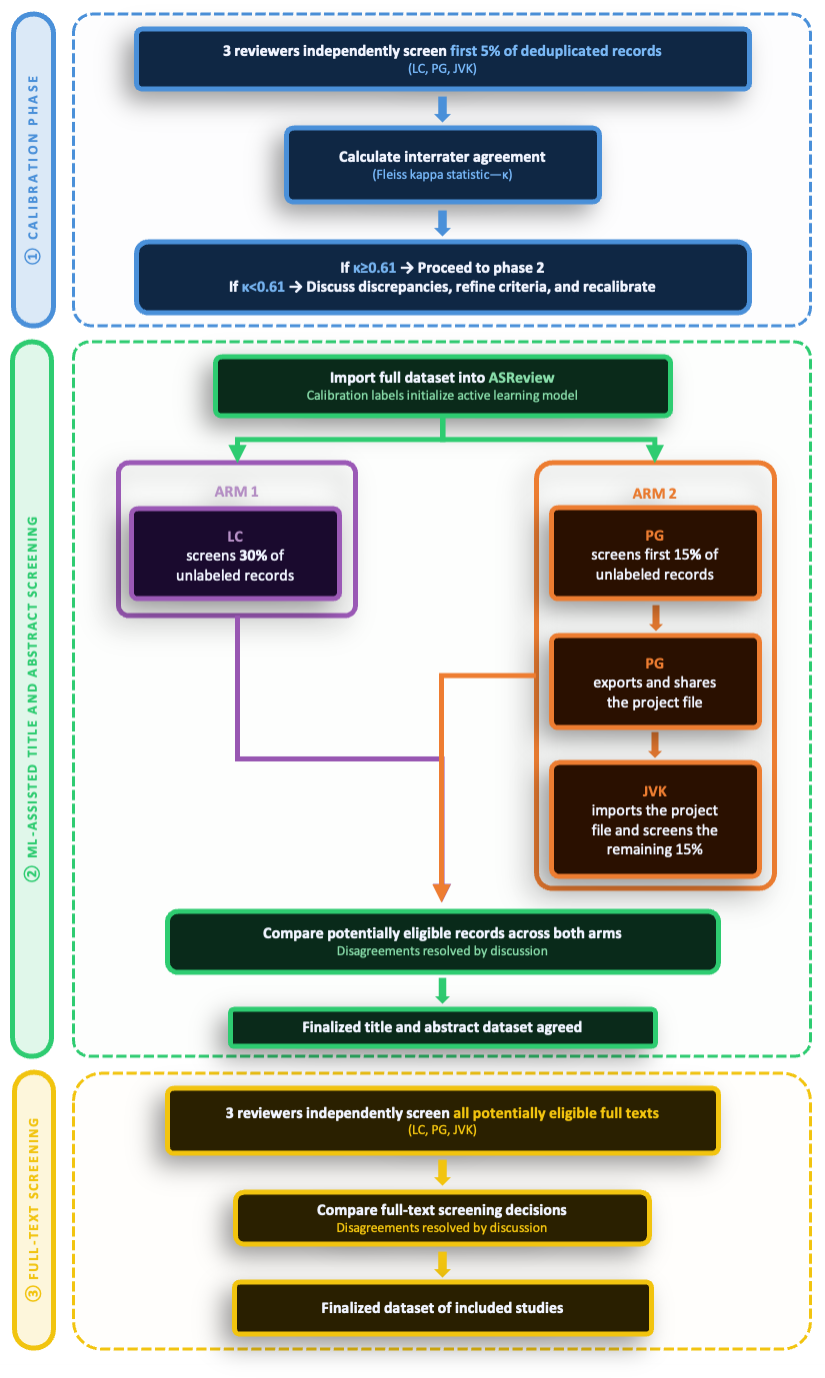
Study selection process. ML: machine learning.

The study selection process will be conducted in 3 sequential stages (Figure 1): an initial calibration phase, a machine learning (ML)–assisted title and abstract screening phase, and a full-text screening phase.

First, the 3 reviewers (LC, PG, and JVK) will independently screen the titles and abstracts of the first 5% (353/7052) of records [20,21] retrieved after deduplication using the Microsoft Excel file generated from Zotero. Following independent screening, decisions will be compared, and interrater reliability will be quantified using the Fleiss κ statistic [22]. A κ value of 0.61 or more will be considered indicative of substantial agreement prior to proceeding to the main screening phase [23,24]. In the case of a κ value less than 0.61, discrepancies will be discussed, eligibility criteria will be further clarified, and an additional calibration round will be conducted if necessary. Any remaining disagreements will be resolved through consensus within the full author team.

In light of the large number of retrieved records and the very low proportion of studies that typically meet eligibility criteria in systematic reviews—on average, below 3% of screened records [25]—the main title and abstract screening phase will be supported by an ML tool to improve efficiency. ASReview [26] is an open-source software that supports reviewers in screening by ranking records according to their predicted likelihood of relevance. All inclusion and exclusion decisions remain the sole responsibility of the human reviewer. The ML tool does not screen records autonomously but assists by determining the order in which records are presented to the reviewer. ASReview learns from each decision a reviewer makes: each time a record is labeled as “relevant” or “irrelevant,” the tool updates its internal model and reorders the remaining records accordingly, so that those most likely to be relevant are prioritized and presented earlier in the screening process.

Given that ASReview does not implement a built-in stopping rule, screening will follow a sampling-based stopping criterion. Empirical evidence indicates that screening the top 25% of records ranked by predicted relevance identifies nearly all studies that would otherwise be included through complete manual screening of the full dataset [27]. To further enhance robustness and reduce the risk of missing relevant records, screening will continue until 30% (2010/6699) of the unlabeled records have been reviewed.

At the operational level, screening will be conducted across 2 parallel screening arms. Both LC and PG will install ASReview locally on their own devices and import the full deduplicated dataset. The records labeled during the calibration phase will serve as prior knowledge to initialize the active learning model in both screening arms, allowing the software to begin ranking the remaining unlabeled records by predicted likelihood of relevance from the outset [26].

In the first screening arm, LC will independently screen 30% (2010/6699) of the unlabeled records. In the second screening arm, PG will screen the first 15% (1005/6699) of the unlabeled records on his own device. Once PG has completed his portion, he will export the ASReview project file and share it with JVK, who will install ASReview on her own device, import the project file, and continue screening from where PG stopped. PG and JVK will thus collectively screen 30% (2010/6699) of the unlabeled records, the same proportion covered independently by LC. Because each reviewer’s independent labeling decisions generate distinct relevance rankings from the same dataset, conducting screening across 2 parallel arms provides an additional safeguard against missing relevant records.

At the end of the title and abstract screening phase, the reviewers will compare the records identified as potentially eligible across both screening arms. Disagreements will be resolved through discussion and, if necessary, through consultation with the remaining members of the author team (JZ, JCZ, DdP, and ADS).

Full-text screening will then be conducted independently by the same 3 reviewers (LC, PG, and JVK) on all records identified as potentially eligible at the title and abstract stage. Inclusion and exclusion decisions will be made in accordance with the predefined criteria described above. Discrepancies arising during full-text screening will be resolved through discussion and, where required, adjudication by the remaining members of the author team (JZ, JCZ, DdP, and ADS).

#### Data Collection Process

Data extraction will be conducted by 3 reviewers (LC, PG, and JVK). Following the completion of full-text screening and the establishment of the final set of included studies, these will be divided equally among the 3 reviewers for independent data extraction. Each study will be extracted by a single reviewer. Upon the completion of full data extraction, a random sample of extracted studies will be independently checked by 1 of the other 2 reviewers not involved in the original extraction of that study (LC, PG, and JVK). Any discrepancies identified during this verification step will be resolved through discussion and, where necessary, through consultation with the remaining members of the author team (JZ, JCZ, DdP, and ADS).

An ad hoc data extraction table will be developed in Microsoft Excel in alignment with the data items described in the following section. The table will be structured to ensure systematic, transparent, and consistent capture of all predefined variables across included studies. Prior to full data extraction, the table will be piloted independently by the 3 reviewers on a subset of included studies to assess clarity, completeness, and feasibility of application. Where necessary, minor refinements will be made to improve usability and ensure appropriate alignment between the predefined data items and the information reported in the studies.

Uncertainties or ambiguities encountered during data extraction will be discussed among the 3 reviewers. If consensus cannot be reached, discrepancies will be resolved through consultation with the remaining members of the author team (JZ, JCZ, DdP, and ADS).

### Data Items

Data will be collected at the level of the individual study. When a single study reports findings for multiple patient-facing DHAs, each DHA will be extracted as a separate entry in the data extraction table to ensure that data are extracted distinctly for each application. Conversely, when multiple studies refer to the same DHA, data will be extracted separately for each study, with the possibility of synthesizing findings across studies referring to the same application where relevant.

The data items are designed to capture how adherence to patient-facing DHAs is conceptualized, operationalized, and measured, along with key characteristics of the applications and their regulatory or implementation context. Extracted information will include study characteristics, features of the DHA, contextual information relevant to regulation or reimbursement, conceptual definitions of adherence and related constructs, as well as operationalization and measurement approaches.

The data extraction framework presented in Table 1 reflects the preliminary set of predefined items developed to address the review objectives. This framework may be refined following pilot testing to ensure conceptual clarity and the adequate capture of relevant information.

**Table 1.** Data extraction table.

Section and data item	Description
A. Study identification	
Study ID	Unique identifier assigned by reviewers
Author(s)	Names of authors
Title	Title of the study
Year of publication	Official year of publication
Country or countries	Country or countries where the study was conducted
Study design	Study design as reported by the authors (eg, RCT^a^, observational study, cohort study, cross-sectional study, qualitative study, mixed methods study, feasibility or pilot study, implementation study, and others)
B. DHA^b^	
Name of DHA	Name of the patient-facing DHA (if reported)
Type of DHA	Functional or regulatory classification as indicated by the authors (eg, digital therapeutic, disease management application, monitoring application, preventive application, and hybrid model)
Delivery mode	Technological format of the application (eg, mobile app, web-based platform, hybrid app or web solution, and other digital interface)
Primary health purpose	Main intended clinical or behavioral objective of the DHA (eg, prevention, monitoring, treatment, rehabilitation, behavior change, multiple objectives if stated)
Therapeutic area	Disease area or health domain addressed by the DHA (eg, diabetes, depression, cardiovascular rehabilitation, and smoking cessation)
C. Regulatory and implementation context	
Regulatory status	Regulatory status of the DHA, including whether it is subject to formal assessment, certification, or approval (if reported)
Reimbursement status	Reimbursement arrangement associated with the DHA (eg, statutory or public reimbursement, private reimbursement, not reimbursed, and not specified)
Health care setting	Clinical and organizational context in which the patient-facing DHA is implemented, including whether it is integrated into routine health care (eg, primary care, specialist care, and hospital-based pathway), delivered in a clinically supervised home-based program, or deployed in a direct-to-consumer setting
D. Adherence conceptualization, operationalization, and measurement	
Explicit conceptual definition	Verbatim statement defining adherence, where provided
Intended or recommended pattern of use	Statement describing the expected or recommended use of the DHA
Operational definition	Rule, formula, or calculation used to quantify or classify adherence
Behavioral metric	Usage indicators used to measure adherence (eg, logins, sessions, time spent, days active, module completion, symptom entries, and composite scores)
Temporal framing	Time reference applied in the measurement of adherence (eg, per day, per week, and across total intervention period)
Threshold or classification rule	Explicit cut-off or categorization criterion used to define adequate adherence (eg, ≥70% module completion). If no threshold is specified, this will be recorded.
Justification for threshold or classification rule (if provided)	Empirical, theoretical, regulatory, or technical rationale provided in support of the selected threshold or classification rule. Presence or absence of justification will be recorded.
Data source	Source of adherence data (eg, application log data, backend analytics, self-report questionnaires, administrative data, mixed sources)

a RCT: randomized controlled trial.

b DHA: digital health application.

### Synthesis Methods

The findings of this scoping review will be synthesized using a narrative synthesis approach, consistent with its exploratory and mapping objectives. Descriptive summaries will be structured according to the main dimensions of the data extraction framework. Where appropriate, simple descriptive statistics (eg, frequencies and proportions) will be used to summarize patterns across studies.

Given the exploratory nature of the review, the expected heterogeneity in study designs and measurement strategies, and the focus on conceptual and methodological mapping rather than effectiveness evaluation, we will not conduct a quantitative synthesis or meta-analysis. Consistent with established guidance for scoping reviews, we will not conduct a formal assessment of the risk of bias or the methodological quality of individual studies [16].

### Dissemination Plan

Preliminary results of this scoping review will be presented at relevant scientific conferences to facilitate early dissemination and community engagement. In addition to the scientific publication, the full data extraction table will be made publicly available in an accessible and navigable format on the DEKODE (*Digital Health Adhärenz: Konzept und datenbasierte Evidenz*) [28] project website to facilitate transparency and support researchers, clinicians, policymakers, and other stakeholders seeking to engage with the underlying evidence. This dissemination strategy is intended to maximize both the scientific impact and the practical accessibility of the review’s findings.

## Results

**Figure 2.**
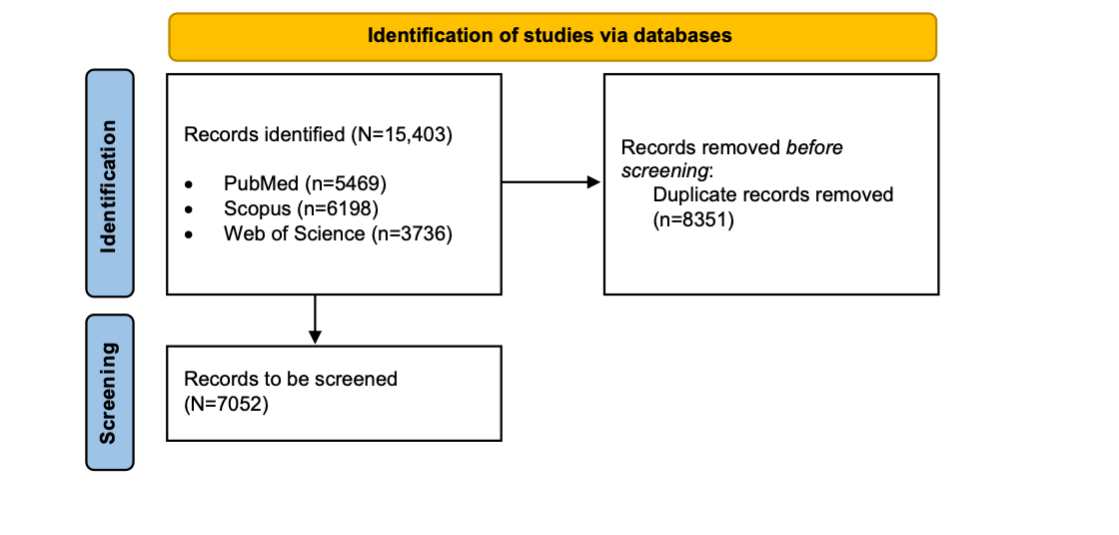
PRISMA (Preferred Reporting Items for Systematic Reviews and Meta-Analyses) 2020 flow diagram.

The database searches conducted on February 26, 2026, identified 15,403 records in total, including 5469 from PubMed (MEDLINE), 6198 from Scopus, and 3736 from Web of Science. After automatic deduplication in Zotero, 7052 records were exported to a Microsoft Excel file for title and abstract screening. As this protocol was prepared before the completion of the screening process, the PRISMA (Preferred Reporting Items for Systematic Reviews and Meta-Analyses) flow diagram (Figure 2) currently reports the identification and deduplication stages only. The complete PRISMA flow diagram will be provided in the final review. The full results are intended to be submitted to a peer-reviewed journal by the end of 2026.

## Discussion

### Main Considerations

This scoping review is conducted as part of the DEKODE project, funded by the Innovation Fund of the German Federal Joint Committee (*Gemeinsamer Bundesausschuss*). The overarching objective of DEKODE is to develop a comprehensive adherence toolkit for patient-facing DHAs, and specifically for DiGAs, that presents adherence factors from the perspective of all relevant stakeholders, including patients, health care providers, manufacturers, and health insurance funds, and outlines strategies to increase evidence-based adherence. To support the early stages of this objective, the project also aims to develop and test measurement criteria and standards for adherence, and to derive implementation recommendations for regulation and medical practice that reward adherence-promoting initiatives.

Achieving these objectives requires, as a necessary foundation, the establishment of a robust conceptual and empirical framework for adherence in the context of patient-facing DHAs. This framework is being developed through 2 complementary literature reviews. This scoping review constitutes the first component, mapping how adherence is currently conceptualized, operationalized, and measured across patient-facing DHAs, and examining whether these approaches differ between applications embedded within formal regulatory or reimbursement frameworks and those operating outside such settings. The second review will focus specifically on the demographic and structural factors that may influence adherence to patient-facing DHAs at the patient, disease, and intervention levels. Together, these 2 reviews are intended to provide the conceptual and empirical grounding that will inform the design of qualitative interviews with patients, health care providers, DiGA manufacturers, and health insurance fund representatives, as well as the development and validation of quantitative survey instruments planned for later stages of the project.

While the DEKODE project is primarily oriented toward the German health care context and the DiGA framework, this review applies no geographical restrictions and encompasses patient-facing DHAs irrespective of their regulatory or reimbursement setting. This deliberate choice reflects the view that a general, global understanding of how adherence is conceptualized, operationalized, and measured across diverse contexts is a necessary precondition before narrowing the focus to the specific German setting. Indeed, efforts to harmonize the assessment of DHAs across European countries, which have been initiated over recent years, reflect the timeliness of an international approach [29]. Furthermore, establishing this broader evidence base first allows for a more grounded and rigorous contextualization of the DiGA-specific findings within the international landscape. At the same time, the insights generated may serve as a foundation for country- or system-specific analyses in other national or regulatory settings.

### Limitations

Several limitations of this review should be acknowledged. First, the use of an ML-assisted screening approach introduces a degree of methodological uncertainty. Although the sampling-based stopping criterion adopted is supported by empirical evidence, it cannot fully exclude the possibility that a small number of eligible studies may be missed. However, acceptable accuracy thresholds may vary depending on the type of review, its research question, and the intended use of its findings. In scoping reviews, the influence of the small residual proportion of unidentified studies beyond the active learning stabilization point is likely to be comparatively limited relative to evidence syntheses focused on quantitative aggregation or comparative effectiveness. Moreover, a residual degree of screening error may also occur in fully manual screening processes, particularly in large-scale reviews involving several thousand records, where reviewer fatigue may affect consistency over time. The conduct of screening across 2 independent parallel arms was intended to mitigate these risks, although residual uncertainty remains inherent in any screening approach. The ASReview workflow was described in detail to maximize methodological transparency and reproducibility. However, ML-assisted screening based on active learning does not guarantee fully deterministic reproducibility of record prioritization across different runs or reviewer interactions, as residual variability may arise from the dynamic and iterative nature of the active learning process. Detailed reporting of model configuration, stopping criteria, prior knowledge settings, and screening procedures may nonetheless support procedural transparency and facilitate methodological replication.

Second, restricting the search to 3 major databases—PubMed (MEDLINE), Scopus, and Web of Science—may result in some relevant literature not being captured. The inclusion of targeted gray literature searches and backward citation tracking is intended to partially offset this limitation.

Third, the temporal restriction to publications from 2020 to 2025, while methodologically justified, means that potentially relevant earlier literature is not systematically captured.

## Supplementary material

10.2196/95287 Checklist 1 PRISMA-P checklist.

10.2196/95287 Checklist 2 PRISMA-ScR checklist.

## Abbreviations

DEKODE: *Digital Health Adhärenz: Konzept und datenbasierte Evidenz*
DHA: digital health application
DiGA: *Digitale Gesundheitsanwendungen*
ML: machine learning
PRISMA: Preferred Reporting Items for Systematic Review and Meta-Analyses
PRISMA-P: Preferred Reporting Items for Systematic Review and Meta-Analyses—Protocols
PRISMA-ScR: Preferred Reporting Items for Systematic Reviews and Meta-Analyses Extension for Scoping Reviews
WHO: World Health Organization
